# Variability of single bean coffee volatile compounds of Arabica and robusta roasted coffees analysed by SPME-GC-MS

**DOI:** 10.1016/j.foodres.2018.03.077

**Published:** 2018-06

**Authors:** Nicola Caporaso, Martin B. Whitworth, Chenhao Cui, Ian D. Fisk

**Affiliations:** a Division of Food Sciences, University of Nottingham, Sutton Bonington Campus, Loughborough LE12 5RD, UK; b Campden BRI, Chipping Campden, Gloucestershire GL55 6LD, UK; c University College London, London, UK

**Keywords:** Single coffee bean, SPME-GC/MS, Headspace analysis, Coffee roasting, Coffee aroma, Coffee volatile compounds, *Coffea arabica* L., *Coffea canephora* L

## Abstract

We report on the analysis of volatile compounds by SPME-GC-MS for individual roasted coffee beans. The aim was to understand the relative abundance and variability of volatile compounds between individual roasted coffee beans at constant roasting conditions. Twenty-five batches of Arabica and robusta species were sampled from 13 countries, and 10 single coffee beans randomly selected from each batch were individually roasted in a fluidised-bed roaster at 210 °C for 3 min. High variability (CV = 14.0–53.3%) of 50 volatile compounds in roasted coffee was obtained within batches (10 beans per batch). Phenols and heterocyclic nitrogen compounds generally had higher intra-batch variation, while ketones were the most uniform compounds (CV < 20%). The variation between batches was much higher, with the CV ranging from 15.6 to 179.3%. The highest variation was observed for 2,3-butanediol, 3-ethylpyridine and hexanal. It was also possible to build classification models based on geographical origin, obtaining 99.5% and 90.8% accuracy using LDA or MLR classifiers respectively, and classification between Arabica and robusta beans. These results give further insight into natural variation of coffee aroma and could be used to obtain higher quality and more consistent final products. Our results suggest that coffee volatile concentration is also influenced by other factors than simply the roasting degree, especially green coffee composition, which is in turn influenced by the coffee species, geographical origin, ripening stage and pre- and post-harvest processing.

## Highlights

• Volatile compounds associated with individual roasted coffee beans were analysed by SPME-GC-MS.
• High inter- and intra-batch variability was observed, especially for 3-ethylpyridine.
• Multivariate statistics allowed prediction of coffee origin (species and location).
• Variability of volatiles is wide, with different chemical classes showing diverse behaviour.

## Graphical abstract

**Figure:**
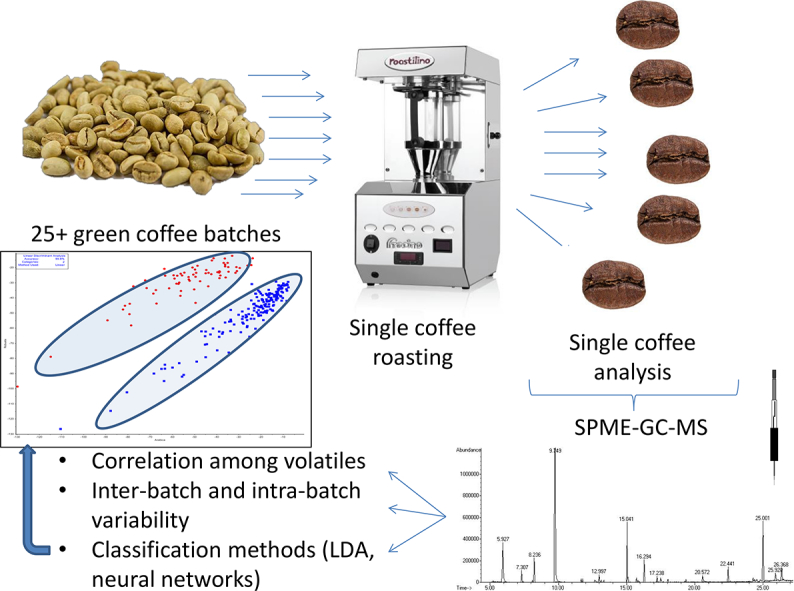

## 1 Introduction

Coffee aroma is one of the most distinctive characteristics of this commodity, which is mostly consumed for its flavour. The concentration of volatile compounds in roasted coffee can undergo dramatic changes depending on the thermal profile applied during the roasting process, but also strongly depends on the green coffee bean composition, the genetic differences in the plant (Sanz, Maeztu, Zapelena, Bello, & Cid, 2002; Tran et al., 2017), seasonal variation within batches (Silva et al., 2005), geographic origin (Freitas & Mosca, 1999), pre- and post-harvest processing of the beans, i.e. wet or dry-processing (Gonzalez-Rios et al., 2007), environmental factors (Bertrand et al., 2012) as well as the presence of defective beans (Agresti, Franca, Oliveira, & Augusti, 2008; Joët et al., 2010) and ripening stage (Toledo, Pezza, Pezza, & Toci, 2016). The roasting process has a dramatic impact on coffee volatiles, as the time-temperature profile affects aroma composition of roasted coffee (Baggenstoss, Poisson, Kaegi, Perren, & Escher, 2008; Franca, Oliveira, Oliveira, Agresti, & Augusti, 2009; Moon & Shibamoto, 2009; Poisson, Blank, Dunkel, & Hofmann et al., 2016), as well as the extractability of each compound and the brewing method (Caporaso, Genovese, Canela, Civitella, & Sacchi, 2014).

Volatile compounds in roasted coffee are mainly represented by aldehydes, ketones, alcohols, esters, pyrazines, furans, acids, nitrogen-containing compounds and volatile phenolic compounds (Fisk, Kettle, Hofmeister, Virdie, & Kenny, 2012; Grosch, 1998; Schenker et al., 2002; Semmelroch & Grosch, 1996; Toledo et al., 2016). The analysis of coffee aroma compounds can be carried out using a variety of analytical techniques, but solid-phase microextraction (SPME) has been widely applied for sampling of volatiles in several food products including roasted coffee and brewed coffee for many decades (Bicchi, Panero, Pellegrino, & Vanni, 1997). SPME gives advantages of using minimal sample treatment and a realistic measurement of the volatiles released from the food products in the headspace, and when coupled with gas-chromatography/mass spectroscopy (GC-MS) is a valid technique for the analysis of headspace aroma released from food matrices, including roasted coffee (Akiyama et al., 2007; Bertrand et al., 2012; Caporaso et al., 2014; Fisk et al., 2012; Risticevic, Carasek, & Pawliszyn, 2008; Zambonin, Balest, De Benedetto, & Palmisano, 2005), and can be used even in mixtures of other food components to measure the headspace release of volatile compounds (Genovese, Caporaso, Civitella, & Sacchi, 2014).

Fisk et al. (2012) applied several sampling techniques to analyse the volatile compounds in roast and ground coffee, and evaluated 15 key aroma compounds for possible discrimination of coffee samples.

Despite a vast amount of work on coffee aroma analysis, the information available on within-batch variability, i.e. at individual coffee bean level, is limited. Whilst previous research applied methods for on-line measurement of volatile compounds to understand aroma formation during roasting (Gloess et al., 2014), little is known in relation to single bean variability. Hertz-Schünemann, Dorfner, Yeretzian, Streibel, and Zimmermann (2013) and Hertz-Schünemann, Streibel, Ehlert, and Zimmermann (2013) applied resonant laser ionisation time-of-flight mass spectrometry (PTR-MS) for single coffee bean analysis, but the coffee beans were roasted to simulate industrial conditions. Yeretzian, Jordan, Badoud, and Lindinger (2002) used PTR-MS to analyse coffee volatiles during the roasting of small batches of six coffee beans, while a more recent work applied single bean roasting (Yener et al., 2016).

Another area of research is the use of coffee volatiles to differentiate roasted coffees for their geographical origins, or for authenticity purposes related to the species, e.g. discrimination of Arabica vs robusta coffee beans. Despite the relatively ease of visual inspection of green coffee beans, the identification of the roasted beans is more complicated. Recently, de Toledo et al. (2017) used data of coffee volatile compounds published by previous research groups to build statistical models for the discrimination of coffee geographical origins. 2-Methylpyrazine and pyridine were reported as the most effective compounds for the discrimination of coffee geographical origins, explaining 97.3% of the variance, but the discrimination accuracy was lower when cross-validation was applied.

Tran et al. (2017) recently investigated the physical and compositional difference among accessions of coffee beans reporting on the variability among coffee plants, by using the cherries harvested from each plant as one sample (sample size of 25–190 g). 35 of these samples were roasted and analysed for their aroma compounds. A wide range of compositional variation was shown for volatile and non-volatile compounds, for example caffeine ranged from 0.82 to 1.75% and trigonelline from 0.80 to 1.38% (dmb). Similar results were also reported by Caporaso, Whitworth, Grebby, and Fisk (2018a) for caffeine and trigonelline variability in single green coffee beans, demonstrating an even wider range on a single bean basis.

Looking at the “elementary unit” of a commodity can help to understand the aroma formation at a basic level, i.e. considering single coffee beans. Previous works trying to analyse very small batches of just a few beans described that the volatile composition changed significantly when looking at single coffee beans compared to batch roasting (Yeretzian et al., 2002). However, no information has been published so far on the variability expected for single beans within the same batch and considering the inter-batch variation. Roasted coffee aroma variability has been mostly investigated in relation to different roasting degrees, with limited focus on the single bean variation when the roasting conditions are constant.

In addition, origin identification of coffee is an interesting topic, due to the price difference between Arabica and robusta species, as well as in terms of authenticity for geographical origin identification, but it remains unclear whether the sole analysis of volatile compounds allows coffee classification.

Therefore, the aim of this article was to study the variability of coffee volatile compounds from a wide range of samples, using SPME-GC-MS as the analytical technique. In addition, classification models were built to understand whether volatile compounds can be effectively used to discriminate single coffee beans according to their origin.

## 2 Materials and methods

### 2.1 Samples and reagents

Samples of commercial green coffee were sourced from UK and European importing companies to obtain a wide geographical distribution of samples. 25 coffee batches were used, belonging to both Arabica and robusta species. Ten green coffee beans were randomly selected from each batch for roasting and analysis, thus the sample number was 250. Their countries of origin are Brazil, Colombia, Costa Rica, Ethiopia, Guatemala, Honduras, India, Kenya, Mexico, Nicaragua, Rwanda, Uganda and Vietnam. Reference chemical compounds were obtained from Sigma-Aldrich (Steinheim, Germany), and Fluka (Buchs, Switzerland).

### 2.2 Coffee roasting

Samples were roasted using a Fracino Roastilino (Birmingham, UK) fluidized-bed roaster. In this system, the beans are moved by a relative high flow of hot air from the bottom of the machine. The roasting conditions applied were isothermal at a set temperature of 210 °C for 3 min (the temperature measured over roasting period had coefficient of variation of 1.8%). Each coffee bean was roasted individually, ground using a manual grinder (Devo, Holland), which was cleaner after each grinding operation using expanded polyethylene, and cleaned the grinder using a brush. Ground coffee samples were immediately stored in a 1.5 mL Eppendorf tube at −80 °C until the moment of analysis.

### 2.3 Analysis of volatile compounds

Coffee volatile compounds were analysed by SPME-GC-MS according to the method of Franca et al. (2009). Exactly 100 mg of ground roasted coffee were weighed and placed in 5 mL vials. Samples were equilibrated for 10 min at 40 °C, followed by 20 min fibre exposure and 5 min injection times. These conditions were chosen according to Ribeiro, Teófilo, Augusto, and Ferreira (2010), using a 1 cm 50/30 μm DVB/Carboxen/PDMS StableFlex fibre (Supelco, Bellefonte, USA). This type of fibre has been previously reported to be the most efficient one for coffee analysis (Akiyama et al., 2007; Risticevic et al., 2008). 3-Heptanone was added as the internal standard, using 20 μL of a 0.01% methanol solution.

The GC conditions were chosen as per Akiyama et al. (2007), slightly adapted to the type of column available. The column used was a 30 m length Zebron ZB-WAX column (Phenomenex, USA), with 0.25 mm internal diameter and 1.00 μm film thickness. GC analysis was performed using a gas chromatograph (Trace Ultra) coupled to a mass spectrometer (PolarisQ, ThermoElectron, San Jose, CA), an RTX-5MS column (5% diphenyl, 95% dimethyl polysiloxane) 30 m 0.25 mm I.D. (Restec, Ireland), and helium as the carrier gas (1 mL min^−1^). The GC injector was operated at 250 °C in the splitless mode, and the GC oven operated at a constant flow of 1.6 mL min^−1^. The GC oven program was set as follows: 40 °C held for 5 min, the followed by an increase to 180 °C at a rate of 3 °C min^−1^; then the rate was set at 10 °C min^−1^ until the temperature reached 250 °C, which was held for 5 min. The ion source (detector) and interface temperatures were 300 °C and 275 °C, respectively. Mass spectra were acquired in the electron impact mode at 70 eV, using m/z range of 50–350 and 2 s scan time. Compound identification was done by comparison of the mass spectra against a database (NIST), when reference compounds were not available. In addition, the identification was carried out by comparing the linear retention indices (LRI) of volatiles under the experimental conditions reported above, with data from the literature. The results were expressed as relative percentage of each compound peak area to the total GC-MS peak area. Each analysis was carried out in duplicate.

### 2.4 Statistical analysis

The standard deviation and coefficient of variation (CV%) were used to describe the variation observed within and between batches. In addition, correlation among volatile compounds was analysed using Pearson correlation and by cluster analysis using the absolute correlation and the complete linkage method to build the clusters. In order to verify whether the intra-batch variability was significant considering the inter-batch variability, ANOVA was performed to check whether multiple groups of samples have the same population mean, at p < 0.05. The Kruskal-Wallis H-test was also performed, which is valid when inter-batch variances are not equal. Data from volatile compounds were also used to understand whether it was possible to discriminate the origin of single roasted coffee beans. Classification of samples was performed using Linear Discriminant Analysis (LDA), and Multiple Layer Perceptron (MLP). For MLP, the whole dataset was segmented into a training set (90% of the samples) and test set (10% of the samples). The resulting model was developed purely on the training set and then the performance of the classifier was evaluated on the test set. The segmentation-training-evaluation process was repeated 10 times to determine the averaged cross-validation accuracy. First, all input attributes (data on volatile compounds) were pre-processed by mean-centering followed by standardisation. Pre-processed data were then forwarded into a 5-layer (3 hidden layers) MLP classifier, with a sigmoid activation function. The structure of the MLP was (26-12-6-2-4), 26 being the dimension of input attributes, and 4 the number of classes to be predicted. The parameters of the MLP were randomly initialized near zero and then tuned by limited memory BFGS (Broyden–Fletcher–Goldfarb–Shanno) optimiser.

## 3 Results and discussion

### 3.1 Coffee aroma volatile composition

Green coffee beans from a wide range of locations and genotypes were individually roasted using an isothermal heating profile, and their volatile profile was analysed by SPME-GC-MS. This is the first time that a SPME-GC-MS-based analysis method has been applied at single coffee bean level to understand variability in this way. The measured average weight loss for the coffee beans was 15.3 ± 2.67%. This corresponds to a medium level of roasting according to Franca et al. (2009), which has been described as suitable to develop the highest content of coffee aroma compounds (Schenker et al., 2002).

**Fig. 1:**
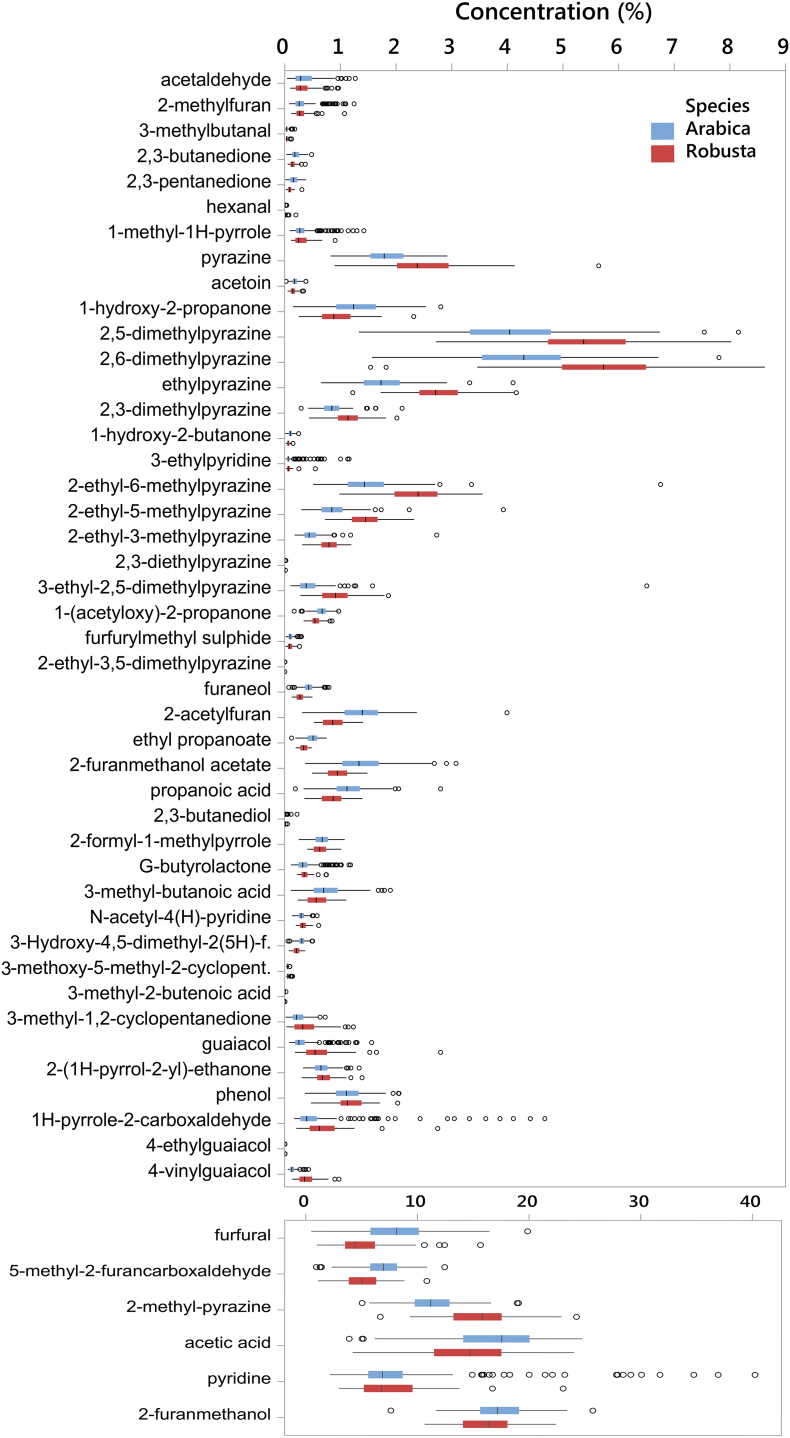
Boxplot distribution of volatile compounds in single roasted coffee beans, by separately showing Arabica and robusta species. Compounds are shown in order of elution (Table 1), except the most abundant ones, shown separately. Vertical bars indicate the median for each compound, horizontal bars indicate the maximum and minimum value, circles indicate possible outliers. The bottom plot shows the most concentrated compounds.

Table 1 lists the compounds identified in the roasted coffee bean headspace. 50 compounds were identified, 21 of which are described in the literature as potent odorants in coffee. Fig. 1 shows the distribution of volatile compounds in single roasted coffee beans in Arabica and robusta samples. 2-Furanmethanol, acetic acid and 2-methyl pyrazine were the most abundant compounds, and Arabica coffees showed higher abundance compared to robusta coffee beans for the first two compounds but not for 2-methyl pyrazine. Pyridine showed the largest range of concentration, and had a similar mean value between the two species, however a wider spread was observed for Arabica, with some samples showing very high pyridine content. Pyrazines were generally higher in robusta, e.g. 2-methyl-pyrazine, 2,6-dimethylpyrazine, 2,5-dimethylpyrazine, pyrazine, ethylpyrazine, 2-ethyl-6-methylpyrazine, 2-ethyl-5-methylpyrazine and 3-ethyl-2,5-dimethylpyrazine. In contrast, compounds such as furfural, 1-(acetyloxy)-2-propanone, 2-acetylfuran, ethyl propanoate, furaneol, 2,3-butanediol, acetoin and 1-hydroxy-2-butanone were found at higher concentration in Arabica coffees. Differences observed among Arabica and robusta coffees are generally supported by previous literature data. Arabica is known to contain higher concentrations of 2,3-butanedione, 2,3-pentanedione and 3-methylbutanal than robusta; and robusta was previously shown to have higher levels of phenols, 1-methylpyrrole and 2,5-dimethylpyrazine (Blank, Sen, & Grosch, 1991).

**Table 1:** Identification of volatile compounds in roasted Arabica and robusta coffee samples analysed by SPME-GC-MS at a single bean level.

n	RT⁎	LRI	Compound	Sensory descriptors	Literature LRI	Odour threshold (ppb)	IM	Chemical group
1	1.29	<1040	**Acetaldehyde**	Pungent, fruity	690	80	MS	Aldehyde
2	1.79	<1040	2-Methylfuran	Pungent, fruity	838-866	4000	MS, L	Furan
3	2.09	<1040	**3-Methylbutanal**	Fruity, malty	906-914	1.2	MS	Aldehyde
4	2.65	<1040	**2,3-butanedione**	Buttery	955	15	MS, L	Ketone
5	3.77	1046	**2,3-Pentanedione**	Buttery, oily, caramel-like	1053-1056	30	MS, ST, L	Ketone
6	4.12	1069	**Hexanal**	Green, grassy, fruity	1024-1087	5	MS, ST	Aldehyde
7	5.24	1128	**1-Methyl-1H-pyrrole**	Smoky, woody, herbal	1123	37	MS, ST	Heterocyclic N
8	6.13	1166	Pyridine	Sour, putrid, fishy, amine, bitter, roasted	1195-1183	2000	MS, L	Heterocyclic N
9	6.95	1201	Pyrazine	Cooked spinach, rancid peanuts, strong	1192-1214	177000	MS, L	Pyrazine
10	8.40	1252	**2-Methyl-pyrazine**	Nutty	1260-1282	60	MS, ST	Pyrazine
11	8.98	1272	**3-Hydroxy-2-butanone**	Sweet, buttery, creamy	1265	800	MS, ST, L	Ketone
12	9.42	1287	Acetol	Sweet, caramellic	1294, 1208	100000	MS	Ketone
13	10.03	1308	**2,5-Dimethylpyrazine**	Nutty, roasted, grassy	1316	2600	MS, ST, L	Pyrazine
14	10.22	1314	**2,6-Dimethylpyrazine**	Chocolate, cocoa, roasted nuts, fried	1319	3100	MS, ST, L	Pyrazine
15	10.39	1319	Ethylpyrazine	Nutty, peanut, butter	1323-1325	6000	MS, ST	Pyrazine
16	10.8	1333	**2,3-Dimethylpyrazine**	Nutty, roasted	1335	250	MS, ST, L	Pyrazine
17	11.66	1361	1-Hydroxy-2-butanone	Sweet, coffee	1368		MS	Ketone
18	11.72	1362	3-Ethylpyridine	Tobacco, oak, moss, leather	1376, 1397		MS	Heterocyclic N
19	11.99	1371	**2-Ethyl-6-methylpyrazine**	Flowery, fruity, hazelnut-like	1363-1381, 1387-1388	30	MS, ST, L	Pyrazine
20	12.12	1375	**2-Ethyl-5-methylpyrazine**	Coffee-like	1393-1395	100	MS, ST, L	Pyrazine
21	12.58	1390	**2-Ethyl-3-methylpyrazine**	Nutty, peanut	1405-1407	130	MS, ST, L	Pyrazine
22	13.57	1422	2,3-Diethylpyrazine	Raw, nutty, green pepper	1454, 1444		MS, L	Pyrazine
23	13.85	1431	**3-Ethyl-2,5-dimethylpyrazine**	Earthy, roasted	1435-1470, 1439	1	MS	Pyrazine
24	14.17	1441	Acetic acid	Pungent, vinegar	1435-1459	34000	MS, ST, L	Acid
25	14.58	1454	Furfural	Sweet, woody, almond	1447-1466	3000	MS, ST, L	Aldehyde
26	14.84	1462	Acetoxyacetone	Fruity, buttery, dairy	1454		MS	Ketone
27	15.21	1474	Furfurylmethyl sulphide	Onion, garlic, sulfuraceous	1476-1480		MS	Sulphide
28	15.38	1479	2-Ethyl-3,5-dimethylpyrazine	Earthy, roasted	1450-1466-1469	1	MS, ST	Pyrazine
29	15.55	1485	Furaneol	Caramel, sweet		31	TI	Ketone
30	15.7	1490	2-Acetylfuran	Sweet, balsam, almond, cocoa	1483, 1499	10000	MS, L	Furan
31	16.76	1520	**Ethyl propanoate**	Sweet, fruity, rum, juicy		10	TI	Ester
32	16.96	1527	**2-Furanmethanol acetate**	Ethereal-floral, herbal-spicy	1514	100	MS	Acetate
33	16.99	1528	Propanoic acid	Pungent, acidic, cheesy, vinegar	1531	20000	MS	Acid
34	17.86	1557	**5-Methylfurfural**	Spice, caramel, maple	1551, 1570	6	MS, ST	Aldehyde
35	18.19	1569	2,3-Butanediol	Fruity, creamy, buttery	1580	75000	MS	Alcohol
36	19.13	1600	2-Formyl-1-methylpyrrole	Roasted, nutty	1610-1620-1626, 1618	40	MS	Pyrrole
37	19.25	1604	γ-Butyrolactone	Creamy, oily, fatty, caramel	1602-1615-1643, 1614	1000	MS,	Ketone
38	20.7	1653	2-Furanmethanol	Caramellic, burnt, smoky	1573-1667	2000	MS, ST, L	Alcohol
39	20.96	1662	3-Methyl-butanoic acid	Cheesey, dairy, creamy, fermented	1670-1678-1683	400	MS, ST	Acid
40	22.23	1705	*N*-Acetyl-4(H)-pyridine	(not available)		20	TI	Heterocyclic N
41	22.91	1728	3-Hydroxy-4.5-dimethyl-2(5H)-furanone	Buttery, seasoning-like	1726*-*2203	50	TI	Ketone
42	23.41	1745	3-Methoxy-5-methyl-2-cyclopenten-1-one	(not available)			TI	Ketone
43	23.98	1764	3-Methyl-2-butenoic acid	Green, phenolic, dairy	1776	14000	MS	Acid
44	24.43	1815	3-Methyl-1,2-cyclopentanedione	Spice, caramellic, maple, sweet, burnt	1822	300	MS	Ketone
45	24.89	1848	**Guaiacol**	Phenolic, burnt, smoky	1850-1859	3	MS, ST, L	Phenolic
46	26.25	1961	2-(1H-pyrrol-2-yl)-ethanone	Smoky, spicy	1952	170000	MS	Ketone
47	26.60	1994	Phenol	Phenolic, plastic, rubber, smoky	1996-2051	2400	MS	Phenolic
48	26.77	2012	1H-pyrrole-2-carboxaldehyde	Musty, beefy, coffee	2028-2030		MS	Heterocyclic N
49	26.86	2022	**4-Ethylguaiacol**	Spicy, phenolic, sweet	2020-2024, 2032-2036	50	MS, ST, L	Phenolic
50	28.28	2186	**4-Vinylguaiacol**	Clove	2151-2187-2205-2210	3	MS, ST, L	Phenolic

⁎ RT, retention time; LRI, linear retention index; IM, identification method, MS, mass spectra, ST, using standard, TI, tentative identification, L, literature LRI values. I.S.: Internal standard. Compounds in bold are those considered as potent odourants in roasted coffee, based on literature data. Sensory descriptors are taken from the literature (Akiyama et al., 2007; Caporaso et al., 2014; Czerny & Grosch, 2000; Maeztu et al., 2001; Grosch, 2001). Odour thresholds are taken from a wide range of bibliographical sources (Amanpour and Selli, 2016, Giri et al., 2010; Miyazato, Nakamura, Hashimoto, & Hayashi, 2013; Nishimura & Mihara, 1990; Piccino, Boulanger, Descroix, & Sing, 2014; Puvipirom & Chaiseri, 2012; Semmelroch & Grosch, 1996; Steinhaus & Schieberle, 2007).

**Fig. 2:**
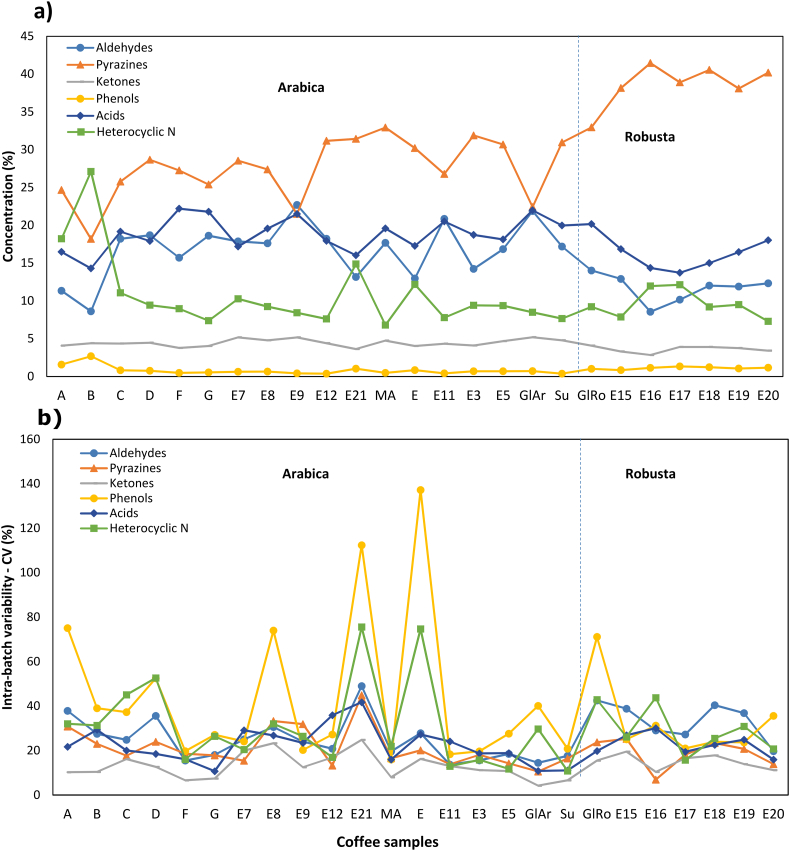
Volatile compounds in roasted coffee beans grouped by chemical classes. The (a) average concentration of each class is shown for each coffee batch (n = 10), and the (b) intra-batch variability is expressed as the relative standard deviation of the ten beans per batch (n = 10).

The average concentration of volatile compounds for each coffee batch is shown in Fig. 2, by grouping the volatiles according to their chemical class and showing each batch separately (10 bean per batch). Pyrazines were often the compounds found at the highest concentration, especially in robusta samples, followed by aldehydes and acids. Heterocyclic nitrogen compounds were less abundant, except for batch “B” (a Rwandan semi-washed Arabica coffee). Ketones and phenols had the lowest concentrations. However, phenols had the highest intra-batch variability in several batches, e.g. A, E8, E21, E, GlRo, but with no clear pattern. The cause of this very variability is unclear, and further investigation is needed. Sample E21 is an Indian Arabica (Monsoon Malabar), which is produced using a particular post-harvesting method, different from the typical wet- or dry-harvest methods. Sample E is a dry-processed Brazilian Arabica. When grouping the volatile compounds according to their chemical classes, strong correlations were also found, particularly between aldehydes and ketones, whose Pearson coefficient was r = 0.752 (p < 0.001). In contrast, the correlation was negative between aldehydes and pyrazine (−0.602, p < 0.001); and between aldehydes and phenols (−0.482, p < 0.001; data not shown).

**Fig. 3:**
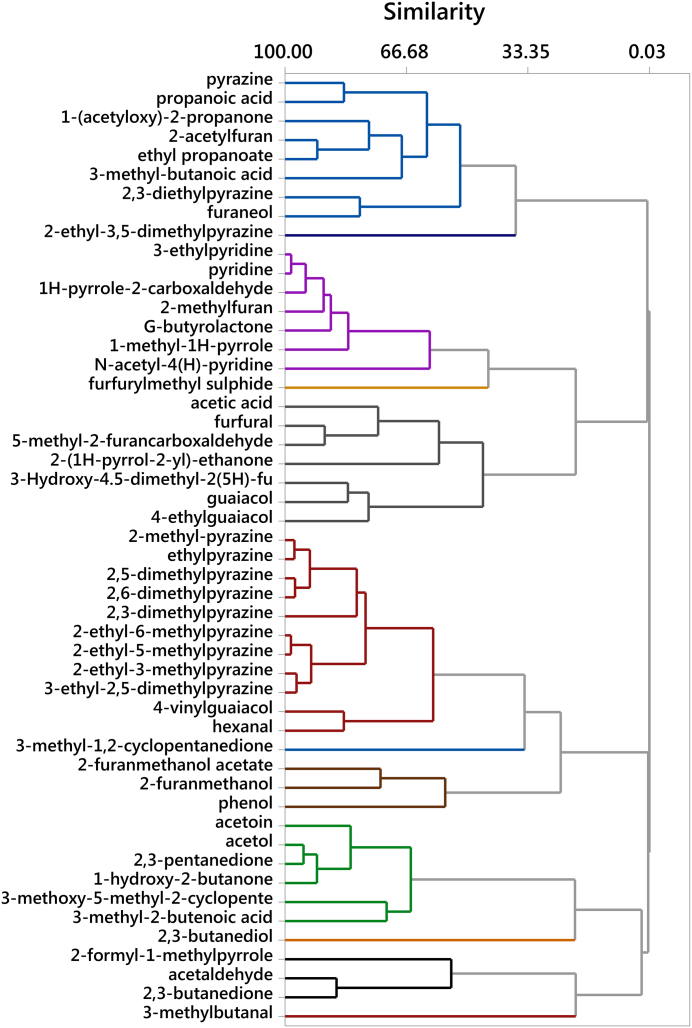
Cluster analysis of volatile compounds in single roasted coffee beans, analysed by SPME-GC-MS (n = 248).

A cluster analysis of all volatile compounds in single coffee beans is shown in Fig. 3, with indication of their correlation. A clear clustering was observed among the majority of pyrazines. This was expected as they originate from the same reaction, also showing similar chemical structure with a few differences in terms of location of the functional groups. Another cluster involving 6 compounds was observed among acetoin, acetol, 2,3-pentandione, 1-hydroxy-2-butanone and other compounds. These compounds are likely to originate from subsequent thermal degradation, for example the strong relationship between 2,3-butanedione and acetoin could be explained by oxidation reactions, these compounds only differing in one functional group. Similarly, the loss of a methyl group in acetoin produces acetol. Other compounds are likely to have a common origin (propanoic acid originating from the loss of a methyl group of 3-methyl butanoic acid), while the formation of other compounds is still unclear and further research is needed to fully understand some of the inter-correlations found herein.

From a Pearson correlation test, several compounds showed a highly significant (p < 0.001) and positive correlation, particularly among pyrazines, with correlation values above 0.8, and some compounds showed up to r = 0.97 (2-ethyl-5-methylpyrazine vs 2-ethyl-6-methylpyrazine, or 2-ethyl-3-methyl pyrazine vs 3-ethyl-2,5-dimethylpyrazine). This result was explained by their common origin from the Maillard reaction, which is strongly dependent on the roasting conditions and the green bean composition. As the thermal conditions were kept constant, differences were attributed to the natural variability of coffee constituents that act as flavour precursors in the Maillard reaction, particularly amino acids and reducing sugars. Thus, in a coffee bean where the limiting reactant is naturally found at higher concentration, higher levels of Maillard reaction products are expected after roasting. Other correlations were found among compounds that share a similar structure and only the position of a functional group is different, or differ in one methyl group, e.g. 1-hydroxy-2-butanone and 1-hydroxy-2-propanone, also correlated with acetoin.

Heterocyclic nitrogen compounds such as pyridine, 1-methyl-1H-pyrrole, 3-ethylpyridine and 1H-pyrrole-2-carboxaldehyde showed some interdependence, as they might originate from different degrees of breakage of the original intermediate molecule. 1-methyl-1H-pyrrole could originate from 3-ethylpyridine when the latter loses a methyl group; the loss of a second methyl group could form pyridine. Similarly, acetoin, 2,3-butanedione and 2,3-pentanedione might originate from the same precursors, and their presence is likely to be due to subsequent cleavage of functional groups. Even acetaldehyde is likely to originate from these three compounds at a later stage of cleavage, which is suggested by the strong correlations found among those compounds.

The Maillard reaction initially gives Amadori products which further degrade into sugar fragmentation products, followed by reactions of dehydration, fragmentation, cyclisation and polymerisation (Van Boekel, 2006). Furfural generation arises from Amadori rearrangement products, in particular from deoxyosones, when the sugar is a pentose. It can be also produced by oxidation of furfuryl alcohol, where the furfuryl alcohol is a product of the reaction of (deoxy)ribose or sucrose with cysteine/methionine (Hertz-Schünemann, Streibel, et al., 2013). In the case of hexoses, hydroxymethylfurfural and 5-methylfurfural are likely to arise from this reaction. Pyrroles, pyranones and furanones are generated from sugar fragmentation of deoxyosones, with a further action of reductions. When other amino acids participate in the reaction, the Strecker reaction of aldehydes with aminoketones, followed by heterocyclisation gives a series of aroma-active volatile compounds, such as pyridines, pyrazines, thiazoles, pyrroles, etc.

Pyridine is a decomposition product of trigonelline, an alkaloid found in the green beans. Guaiacols are generated from caffeic and ferulic acids, which are derivatives of chlorogenic acids (Yeretzian et al., 2002). Pyridine has been previously related to the roasting time: high pyridine concentrations are produced at the initial stage of roasting, which is followed by continuous decrease at longer times (Baggenstoss et al., 2008). However, in the present case the roasting time was uniform for all the samples, thus differences in pyridine concentration have to be attributed to differences in the initial levels of green coffee bean aroma precursors.

Models for formation of 4-vinylguaiacol, guaiacol and phenol during roasting were proposed by Dorfner, Ferge, Kettrup, Zimmermann, and Yeretzian (2003). Degradation of 5-feruloylquinic acid was reported as the origin of melanoidins and phenolic volatile compounds in coffee, due to hydrolysis, polymerisation and oxidation. 4-vinylguaiacol, guaiacol and phenol concentration is strictly interrelated, as ferulic acid degradation generates 4-vinylguaiacol.

Degradation of chlorogenic acids causes the appearance of a wider series of neo-formation products (Kamiyama, Moon, Jang, & Shibamoto, 2015). Phenol and 2,5-dimethylfuran were reported to be formed from 5-caffeoylquinic acid degradation over roasting. 4-vinylphenol was reported as a degradation product of 5-caffeoylquinic acid under slightly acidic conditions. Similarly, furaneol, also named 4-hydroxy-2,5-dimethyl-3(2H)-furanone, has been previously reported in green and roasted coffee (Scheidig, Czerny, & Schieberle, 2007; Yeretzian et al., 2002).

Despite several mechanisms proposed in the literature to explain the generation of alpha-diketones, such as 2,3-butanedione and 2,3-pentanedione, no clear mechanism is accepted, but it is accepted that the formation pathway involves glucose, or an intermediate product of sucrose degradation (Baggenstoss et al., 2008). Previous studies proposed their formation either from sugar degradation or through further interaction of sugar degradation products with amino acids (Yaylayan & Keyhani, 1999), while Baggenstoss et al. (2008) suggested an independent generation.

### 3.2 Variability of volatile compounds

The intra-batch and inter-batch variability of coffee volatile composition is shown in Table 2. The variability is illustrated as the relative standard deviation of 10 beans within each batch, as well as reporting the variation between batches. High intra-batch variability indicates that large differences were observed in beans from the same batch. 3-ethylpyridine, hexanal, 1H-pyrrole-2-carboxaldehyde, 2,3-butanediol, 3-methyl-2-butenoic acid and 3-methyl-2,5-dimethylpyrazine showed a variability above 40% (CV). Other compounds also showed a wide range of concentrations. For example, 2-ethyl-3,5-dimethylpyrazine had an intra-batch CV% of 39%. It should be noted that the variability is expressed as relative standard deviation, therefore the high value found for compounds found at very low concentrations might suffer from an overestimation of the observed variability due to the analytical error of the method, as in the case of some phenols.

**Table 2:** Variability of volatile compounds in roasted coffee beans. Each volatile compound was expressed as relative percentage of the GC peak area (%) on the total peak areas. The within batch bean-to-bean variability was expressed as coefficient of variation (CV%) of the mean for 10 beans per batch; the between bean variability was calculated as CV% of all 248 coffee beans analysed (all 25 batches). The values in brackets indicate the maximum variation observed expressed as the ratio of the maximum and minimum concentration of each volatile compound (as per Tikunov et al., 2005).

Volatile compound	Mean	SD	min	max	range	Variation within batch (n = 10) – CV (%)		Variation between beans (n = 248) – CV (%)	
2-Furanmethanol	17.05	2.66	7.63	25.72	18.09	14.00	(2.4)	15.59	(3.4)
Acetic acid	16.12	4.47	3.9	24.77	20.87	24.54	(3.1)	27.73	(6.3)
2-Methyl-pyrazine	12.51	3.29	5.04	24.24	19.2	18.92	(2.8)	26.26	(4.8)
Pyridine	8.58	5.77	2.17	40.23	38.07	35.99	(3.3)	67.22	(18.6)
Furfural	7.34	3.57	0.5	19.87	19.37	34.57	(4.9)	48.55	(39.8)
5-Methyl-2-furancarboxaldehyde	6.35	2.05	0.95	12.44	11.48	24.17	(3.0)	32.26	(13.0)
2,6-Dimethylpyrazine	4.69	1.42	1.54	11.46	9.91	23.66	(2.5)	30.28	(7.4)
2,5-Dimethylpyrazine	4.47	1.39	1.33	12.85	11.51	23.15	(2.4)	31.19	(9.6)
Ethylpyrazine	2.02	0.68	0.65	4.16	3.51	21.65	(2.2)	33.47	(6.4)
Pyrazine	2.01	0.61	0.82	5.64	4.82	20.50	(2.1)	30.54	(6.9)
2-Ethyl-6-methylpyrazine	1.74	0.72	0.51	6.75	6.24	27.06	(2.7)	41.33	(13.3)
2-Furanmethanol acetate	1.28	0.48	0.37	3.08	2.71	19.73	(2.3)	37.59	(8.3)
2-Acetylfuran	1.25	0.48	0.31	3.99	3.68	23.07	(2.6)	38.07	(12.8)
1-Hydroxy-2-propanone	1.19	0.48	0.15	2.8	2.65	25.73	(3.1)	40.49	(18.5)
1H-pyrrole-2-carboxaldehyde	1.13	0.3	0.36	2.05	1.69	49.12	(5.8)	104.76	(27.9)
Propanoic acid	1.08	0.36	0.19	2.8	2.61	24.32	(3.1)	33.46	(14.7)
2-Ethyl-5-methylpyrazine	1.04	0.45	0.3	3.93	3.63	26.25	(2.6)	42.89	(13.2)
2,3-Dimethylpyrazine	0.94	0.28	0.29	2.11	1.81	20.90	(2.5)	30.36	(7.2)
3-Methyl-butanoic acid	0.72	0.31	0.11	1.9	1.79	26.55	(3.3)	43.69	(17.1)
2-(1H-pyrrol-2-yl)-ethanone	0.68	0.18	0.31	1.39	1.08	17.35	(1.9)	27.28	(4.5)
2-Formyl-1-methylpyrrole	0.66	0.15	0.25	1.08	0.82	15.07	(1.9)	23.22	(4.3)
Phenol	0.66	0.69	0.17	4.67	4.5	16.98	(2.0)	26.70	(5.6)
1-(Acetyloxy)-2-propanone	0.63	0.14	0.17	0.97	0.8	14.77	(2.0)	22.16	(5.8)
3-Ethyl-2,5-dimethylpyrazine	0.60	0.51	0.1	6.5	6.4	43.05	(6.0)	85.35	(63.6)
2-Ethyl-3-methylpyrazine	0.57	0.27	0.18	2.73	2.55	28.26	(3.2)	47.13	(15.4)
Ethyl propanoate	0.45	0.13	0.12	0.75	0.63	16.75	(2.4)	27.78	(6.2)
Guaiacol	0.42	0.33	0.08	2.8	2.72	36.56	(4.9)	77.48	(36.3)
Furaneol	0.39	0.13	0.07	0.79	0.72	18.16	(2.5)	32.81	(11)
γ-Butyrolactone	0.38	0.18	0.11	1.18	1.07	20.70	(2.9)	48.32	(10.7)
Acetaldehyde	0.36	0.23	0.04	1.27	1.23	28.88	(3.8)	63.75	(30.0)
1-Methyl-1H-pyrrole	0.33	0.21	0.09	1.42	1.33	31.63	(4.6)	63.22	(15.8)
2-Methylfuran	0.32	0.2	0.08	1.25	1.17	33.17	(5.9)	62.83	(15.6)
*N*-acetyl-4(H)-pyridine	0.32	0.08	0.13	0.61	0.47	16.47	(2.2)	24.70	(4.6)
3-Hydroxy-4.5-dimethyl-2(5H)-furanone	0.28	0.08	0.06	0.5	0.44	18.93	(3.5)	29.28	(8.5)
3-Methyl-2-butenoic acid	0.28	0.2	0.02	1.23	1.22	43.27	(8.2)	68.87	(75.8)
4-Vinylguaiacol	0.21	0.16	0.05	0.97	0.92	28.39	(3.8)	74.20	(18.8)
2,3-Butanedione	0.19	0.08	0.03	0.48	0.45	19.27	(3.0)	43.08	(17.0)
Acetoin	0.18	0.06	0.02	0.38	0.36	21.41	(4.2)	33.33	(15.8)
2,3-Pentanedione	0.14	0.08	0.01	0.38	0.37	26.37	(5.9)	55.01	(34.8)
3-Ethylpyridine	0.11	0.16	0.02	1.16	1.14	53.29	(11.2)	143.09	(63)
Furfurylmethyl sulphide	0.10	0.05	0.01	0.3	0.28	27.42	(9.4)	51.00	(20.8)
1-Hydroxy-2-butanone	0.09	0.04	0.01	0.25	0.24	27.03	(8.3)	44.32	(29.1)
3-Methoxy-5-methyl-2-cyclopenten-1-one	0.05	0.02	0.02	0.14	0.12	18.73	(6.3)	34.78	(6.8)
3-Methylbutanal	0.04	0.02	0.01	0.18	0.17	34.29	(6.2)	60.90	(26.8)
3-Methyl-1,2-cyclopentanedione	0.01	0.003	0.003	0.02	0.02	15.74	(7.1)	24.91	(6.0)
Hexanal	0.01	0.02	0	0.20	0.2	49.85	(6.4)	135.08	(103.5)
2,3-Butanediol	0.01	0.02	0	0.22	0.22	48.54	(6.0)	179.28	(179.2)
2,3-Diethylpyrazine	0.004	0.002	0	0.019	0.018	28.44	(3.2)	51.26	(15.3)
4-Ethylguaiacol	0.001	0.001	0	0.005	0.005	27.74	(3.1)	48.46	(19.4)
2-Ethyl-3,5-dimethylpyrazine	0.001	0.001	0	0.001	0.001	39.35	(4.9)	54.62	(20.2)

Other compounds such as guaiacol, pyridine, furfural and 3-methylbutanal all had CV% values above 34. Pyridine showed the largest range, from ~2 to 40%. On the contrary, the least variable compounds were generally ketones, with 2-furanmethanol showing the lowest variation within and between batches, which was 14 and 15.6 (CV%), respectively. Considerably higher variation was observed between batches, with several compounds showing variation above 100% CV, 13 compounds having CV above 60%, and another 14 compounds above 40% CV. The highest values for inter-batch variability (CV %) were obtained for 2,3-butanediol (179.3%), 3-ethylpyridine (143.1%) and hexanal (135.1%).

The ratio between the highest and the lowest concentration of each volatile was used as another indicator of the spread of concentrations. This ratio ranged from a minimum of 1.9 for 2-formyl-1-methylpyrrole and 2-(1H-pyrrol-2-yl)-ethanone up to 11.2 for 3-ethylpyridine, when considering the intra-batch variability.

The variability obtained in this study was in line with previous research for other food products (Tikunov et al., 2005; Weingart, Kluger, Forneck, Krska, & Schuhmacher, 2012), however a direct comparison cannot be made as no other work has reported on the single-bean variability of coffee volatiles. Tran et al. (2017) reported on coffee aroma variability in terms of morphology and chemical composition, by roasting 50 g of coffee at 180–185 °C for 4 min, and selecting 18 volatile compounds. The reported CV% values varied from 14% (4-vinylguaiacol) to 62% (geraniol). They reported a strong dependence of 4-ethylguaiacol or guaiacol on the roasting degree, which was assessed by measuring the colour values. In addition, positive correlations were reported between aldehydes and ketones, aldehydes and phenolic compounds, as well as aldehydes and pyrazines.

The results of ANOVA (Table 3) indicate strong and significant differences among coffee samples in terms of volatile compounds, with p < 0.05. This was likely due to the clear difference between Arabica and Robusta coffees, thus ANOVA was independently performed on the samples belonging to the two groups. The ANOVA and H-test were performed on 16 batches of Arabica and 7 batches of robusta separately. For all volatiles in Arabica batches, both the ANOVA and H-test suggest that they varied significantly from batch to batch, except 3-ethyl-2,5-dimethylpyrazine and 2-ethyl-3,5-dimethylpyrazine. However, for robusta, for most volatiles there was no evidence showing that the volatile means of different batches are statistically different. This might, however, be attributed to the lower number of samples used for robusta compared to Arabica batches. Similar results were obtained using ANOVA or H-test, even though these tests are based on different statistical assumptions.

**Table 3:** Results of ANOVA test and H-test on single coffee bean volatile compounds, by reporting the significance value (p). Values indicated in bold are those above 0.05.

	All samples		Arabica		Robusta	
	ANOVA	H-test	ANOVA	H-test	ANOVA	H-test
Acetaldehyde	0.0000	0.0000	0.0000	0.0000	0.0000	0.0000
2-Methylfuran	0.0000	0.0000	0.0000	0.0000	0.0107	0.0039
3-Methylbutanal	0.0000	0.0000	0.0000	0.0000	0.0049	0.0172
2,3-Butanedione	0.0000	0.0000	0.0000	0.0000	0.0000	0.0000
2,3-Pentanedione	0.0000	0.0000	0.0000	0.0000	0.0000	0.0000
Hexanal	0.0000	0.0000	0.0021	0.0000	**0.1338**	**0.0748**
1-Methyl-1H-pyrrole	0.0000	0.0000	0.0000	0.0000	**0.0621**	0.0231
Pyridine	0.0000	0.0000	0.0000	0.0000	0.0318	0.0438
Pyrazine	0.0000	0.0000	0.0000	0.0000	0.0203	0.0040
2-Methyl-pyrazine	0.0000	0.0000	0.0000	0.0000	**0.1778**	**0.0766**
Acetoin	0.0000	0.0000	0.0000	0.0000	0.0021	0.0032
1-Hydroxy-2-propanone	0.0000	0.0000	0.0000	0.0000	0.0008	0.0026
2,5-Dimethylpyrazine	0.0000	0.0000	0.0000	0.0000	**0.0923**	**0.1425**
2,6-Dimethylpyrazine	0.0000	0.0000	0.0000	0.0000	**0.1009**	**0.1113**
Ethylpyrazine	0.0000	0.0000	0.0000	0.0000	**0.1470**	**0.0692**
2,3-Dimethylpyrazine	0.0000	0.0000	0.0000	0.0000	**0.1859**	**0.1593**
1-Hydroxy-2-butanone	0.0000	0.0000	0.0000	0.0000	**0.0613**	**0.0797**
3-Ethylpyridine	0.0000	0.0000	0.0000	0.0000	**0.1299**	**0.0387**
2-Ethyl-6-methylpyrazine	0.0000	0.0000	0.0006	0.0000	0.0316	0.0321
2-Ethyl-5-methylpyrazine	0.0000	0.0000	0.0009	0.0000	0.0280	0.0402
2-Ethyl-3-methylpyrazine	0.0000	0.0000	0.0052	0.0000	0.0106	0.0154
2,3-Diethylpyrazine	0.0000	0.0000	0.0001	0.0000	**0.0584**	**0.0711**
3-Ethyl-2,5-dimethylpyrazine	0.0000	0.0000	**0.1226**	0.0001	0.0100	0.0214
Acetic acid	0.0002	0.0001	0.0056	0.0041	0.0189	0.0302
Furfural	0.0000	0.0000	0.0000	0.0000	**0.2447**	**0.1430**
Acetoxyacetone	0.0000	0.0000	0.0000	0.0000	**0.2639**	**0.2943**
Furfurylmethyl sulphide	0.0000	0.0000	0.0000	0.0000	0.0000	0.0003
2-Ethyl-3,5-dimethylpyrazine	0.0130	0.0120	**0.0568**	0.0223	**0.5241**	**0.5214**
Furaneol	0.0000	0.0000	0.0000	0.0000	0.0000	0.0002
2-Acetylfuran	0.0000	0.0000	0.0000	0.0000	**0.7813**	**0.7157**
Ethyl propanoate	0.0000	0.0000	0.0000	0.0000	**0.5684**	**0.5621**
2-Furanmethanol acetate	0.0000	0.0000	0.0000	0.0000	**0.8584**	**0.8732**
Propanoic acid	0.0000	0.0000	0.0012	0.0001	**0.8021**	**0.8780**
5-Methylfurfural	0.0000	0.0000	0.0000	0.0000	**0.0924**	**0.0708**
2,3-Butanediol	0.0000	0.0000	0.0000	0.0000	**0.0320**	0.0000
2-Formyl-1-methylpyrrole	0.0000	0.0000	0.0000	0.0000	0.0006	0.0023
G-butyrolactone	0.0000	0.0000	0.0000	0.0000	0.0006	0.0007
2-Furanmethanol	0.0001	0.0003	0.0016	0.0014	**0.2632**	**0.3563**
3-Methyl-butanoic acid	0.0000	0.0000	0.0000	0.0000	**0.0648**	**0.0506**
*N*-Acetyl-4(H)-pyridine	0.0000	0.0000	0.0000	0.0000	**0.0351**	0.0167
3-Hydroxy-4.5-dimethyl-2(5H)-furanone	0.0000	0.0000	0.0000	0.0001	0.0281	0.0131
3-Methoxy-5-methyl-2-cyclopenten-1-one	0.0000	0.0000	0.0000	0.0000	0.0000	0.0001
3-Methyl-2-butenoic acid	0.0000	0.0000	0.0000	0.0000	**0.5103**	**0.6271**
3-Methyl-1,2-cyclopentanedione	0.0000	0.0000	0.0000	0.0000	**0.3911**	**0.2981**
Guaiacol	0.0000	0.0000	0.0000	0.0000	**0.2999**	0.0086
2-(1H-pyrrol-2-yl)-ethanone	0.0000	0.0000	0.0000	0.0000	0.0000	0.0000
Phenol	0.0000	0.0000	0.0000	0.0000	0.0000	0.0000
2-Formylpyrrole	0.0000	0.0000	0.0000	0.0000	**0.2701**	**0.0448**
4-Ethylguaiacol	0.0000	0.0000	0.0000	0.0000	0.0260	0.0013
4-Vinylguaiacol	0.0000	0.0000	0.0000	0.0000	0.0000	0.0000

Some coffee volatile compounds were previously correlated to the roasting degree, e.g. 2,3-pentanedione, 2,5-dimethylpyrazine, 2-ethylpyrazine, 2,3-dimethylpyrazine, 3-ethyl-2,5-dimethylpyrazine, guaicol, 2-methylbutanal, 2-ethylguaicol, and 4-vinylguaiacol (Franca et al., 2009; Moon & Shibamoto, 2009; Ribeiro, Teófilo, Salva, Augusto, & Ferreira, 2013; Toci & Farah, 2014). The concentration of total furans and generally compounds originating from chlorogenic acid degradation such as phenols and lactones is therefore expected to be higher at a more intense roasting (Moon & Shibamoto, 2009). However, our samples were roasted under the same time-temperature profile, and the majority of these compounds have been found in the present study to be significantly different (p < 0.05) among the coffee batches, indicating that their concentration is also influenced by other factors rather than simply heat exposure, the levels of volatile precursors in the green bean likely representing the most important factor; this is in turn influenced by the coffee species, geographical origin, ripening stage, pre- and post-harvest processing.

The roasting of Arabica and Robusta coffees can be carried out using different roasting times, but in this experiment all the roasting operations were kept constant for all the samples in order to carry out a better direct comparison. In addition to other coffee constituents, the moisture content of the green coffee beans might be different even within the same batch, as recently reported (Caporaso, Whitworth, Grebby, & Fisk, 2018b), therefore some differences observed in terms of volatile compounds might be attributed to the initial different moisture content. However, as shown in Caporaso et al. (2018b) the range of natural moisture content of coffee beans available to the market is relatively narrow, therefore the little difference in moisture content would not fully explain the large differences found herein. Similarly, there might be influence of the post-harvest processing, i.e. wet and dry processing, which affect the chemical composition of the coffee beans and therefore the formation of volatile compounds.

### 3.3 Classification models for origin discrimination

**Fig. 4:**
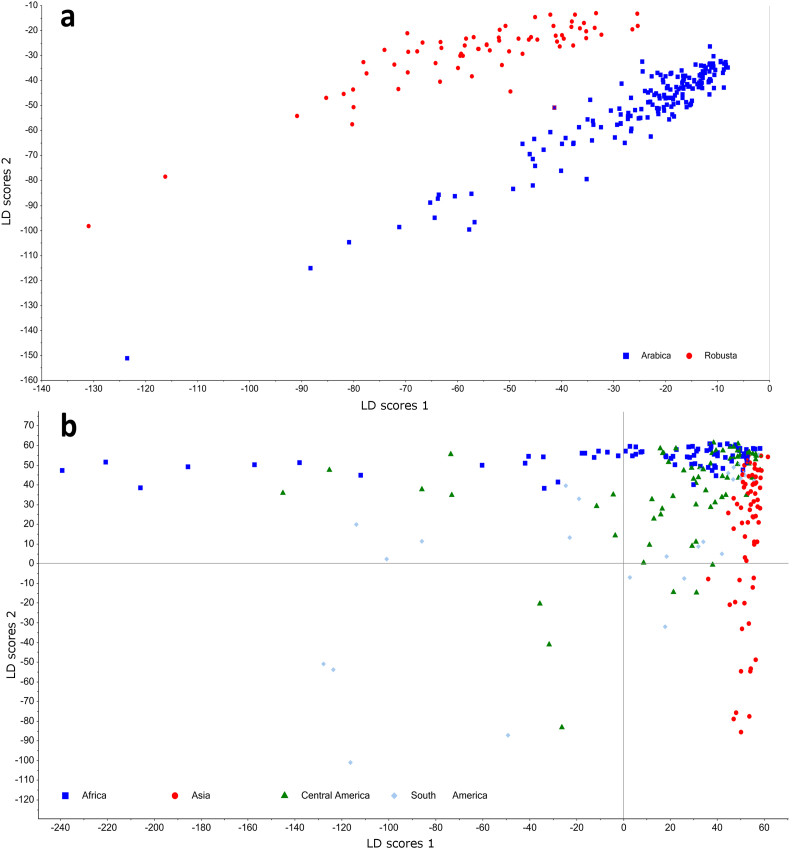
Results of Linear Discriminant Analysis (LDA) applied to discriminate roasted coffee beans according to their (a) botanical species or (b) geographical origin, based on volatile composition assessed by SPME-GC-MS (expressed as % total peak areas). n = 248. Each point represents a sample of a single coffee bean.

Fig. 4 shows the results of Linear Discriminant Analysis (LDA) applied to single coffee beans to discriminate their species (Arabica or robusta) or geographical origin, grouped into four major areas. The PCA explained 79% of the total variance, and limited separation between Arabica and robusta was obtained. On the contrary, excellent separation was obtained by LDA, with an overall classification accuracy of 99.8%. Thus, Arabica and robusta coffee beans can be effectively classified according to their volatile profile despite the large variation found at individual coffee bean level. 4-Ethylguaiacol followed by 2-ethyl-3,5-dimethylpyrazine were the compounds showing the highest scores for the discriminant function. The concentration of the first compound was significantly higher in robusta than Arabica coffees, while the latter compound had lower concentration in robusta then Arabica. However, both compounds were found at very low concentrations, but they are potent odorants as their odour threshold is very low. Other compounds such as ethylpyrazine, 2,3-butanediol, 4-vinylguaiacol and 2,3-pentanedione, had the lowest influence on the classification model, thus suggesting very limited or no significant difference in terms of concentrations between the two species. Previous authors reported quantitative differences between the coffee species, whereas they cannot be strictly considered as molecular markers, e.g. 4-ethylguaiacol, 4-vinylguaiacol and some pyrazines are more abundant in robusta, while 2,3-butanedione and 2,3-pentanedione are more abundant in Arabica (Blank et al., 1991; Semmelroch & Grosch, 1996). From the LDA model, the most important volatiles in discriminating coffee species were mostly pyrazines (2-ethyl-5-methylpyrazine, 2,5-dimethylpyrazine, 2-ethyl-6-methylpyrazine, 2-ethyl-3-methylpyrazine, ethylpyrazine), while other compounds such as 4-vinylguaiacol, 2-hydroxy-2-butanone and 3-ethylpyridine were also strongly contributing to the differentiation.

The LDA model built for geographical origin gave satisfactory results, demonstrating good discrimination ability for the four geographical locations. The overall model accuracy for geographical origin was 95.97% correct classification. A quadratic function gave better classification performance than a linear function (data not shown). The correct classification was 91.3%, 97.1%, 98.6% and 100.0% for the four regions (Africa, Asia, Central America, South America), respectively. The average cross validation error was 11.3%, with the best cross-validation performance obtained for Asia.

A discrimination model was also built to verify whether it is possible to classify coffee beans based on their post-harvest processing, i.e. wet- and dry-processing. The LDA showed 75.6% correct classification for the dry-processed beans and 82.3% for wet-processed ones. The cross-validation gave 68.9 and 80.4% correct classification for dry and wet processed coffees, respectively (data not shown), however this might be due to correlation between origins and processing in some samples.

An alternative statistical approach tested to build classification models was Multiple Layer Perceptron (MLP), which is a class of feed-forward artificial neural network. The final cross-validation accuracy of the MLP model was 91.9%. The confusion matrix for the geographical origin classification model is reported in Table 4. Samples from Asia had the best prediction, with only two mislabelled samples (predicted as Africa). In the case of Africa and Central America, 7 and 6 samples were mislabelled, respectively. The prediction accuracy was thus as follows: Asia, 97.1%; Africa, 98.7%; Central America, 92.7% and South America, 58.6%. The prediction accuracy for all origins was very good except the one for South America, for which we believe it is due to the insufficient training samples in the category.

**Table 4:** Confusion matrix of the classification model for geographical origin prediction for single roasted coffee beans based on their volatile profiles.

		Predicted origin			
		Asia	Africa	Central America	South America
True origin	Asia	68	1	1	0
	Africa	1	79	0	0
	Central America	3	1	64	1
	South America	1	0	11	17

**Fig. 5:**
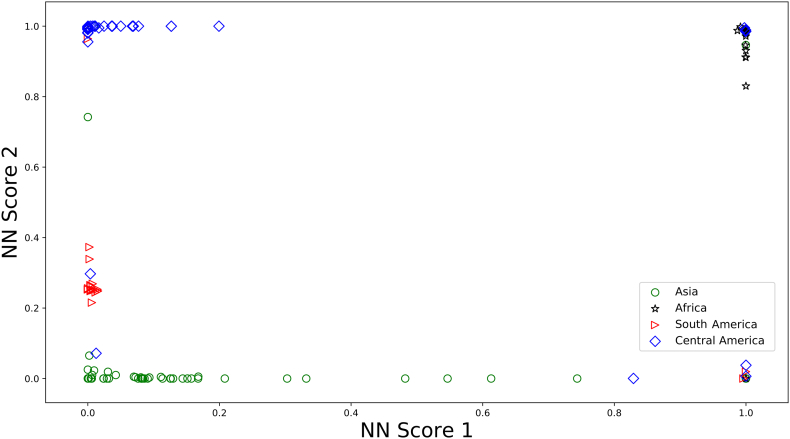
Classification model for coffee origin using Neural Network (NN). NN score 1 and NN score 2 are extracted features from the neural network model.

The last hidden layers of the MLP were extracted as Neural Network (NN) scores and are shown in Fig. 5. The whole MLP model can be viewed as a feature extractor, which maps the original volatile profile onto a two dimensional space, where the final classification was made. Each of the extracted features (NN score 1 and NN score 2) is a combination of all volatile compounds, which most efficiently distinguish coffee samples of various origins. From the Neural Network (NN) scores scatter plot the clustering of the coffee beans from the same origin can be observed.

The use of the relative abundance profiles of the volatile compounds from roasted coffee beans to discriminate the botanical and geographical origin is interesting for the coffee market for quality control purposes and to check coffee bean authenticity, as coffees from different origins can have different market price to reflect differences in perceived quality and availability. The first distinction in the coffee industry is related to the coffee species, Arabica being sold at a price more than double than of robusta coffees. Whilst discrimination of coffee species on the green beans is relatively easy for experts, it becomes more difficult in the roasted coffee, thus the use of SPME-GC-MS analysis of volatile compounds could be a useful means for coffee origin authentication even in mixtures of whole roasted beans, where a representative sample of single coffee beans could be analysed to give indication on the ratio between Arabica and robusta beans.

The successful classification of roasted coffee beans is likely to be related to initial different contents of aroma precursors in the green beans, due to environmental, genetic or processing factors. Previous authors have reported on the differences in volatile compounds of coffees produced using wet- or dry-processing. However, the lack of strong differences between the wet- and dry-processed beans might be due to the presence of other factors affecting aroma compound variability, in particular the intrinsic characteristics of the coffee species and variability of chemical composition in single beans (Gonzalez-Rios et al., 2007).

Contradictory results have been reported in the literature regarding the possibility of using coffee volatile compounds for geographical origin indication. For example, Bicchi et al. (1997) reported successful differentiation of coffees based on their origin using commercially available blends. On the contrary, Zambonin et al. (2005) described the absence of any particular volatile marker related to the geographical origin of the samples. More recently, Bressanello et al. (2017), applied SPME-GC-MS for the analysis of volatile compounds in roasted Arabica and robusta coffee to classify three robusta coffee samples based on their geographical origin, but no clear clustering was obtained except for their Indonesian sample, for the ground coffee and coffee brew. From our results to predict coffee geographical origin, three pyrazines (2-ethyl-5-methylpyrazine, 2,5-dimethylpyrazine and 2-ethyl-3-methylpyrazine) were the most important compounds in the model, followed by 3-ethylpyridine, acetoxyacetone, guaiacol, ethylpyrazine and 2-furanmethanol.

Risticevic et al. (2008) used a sample size similar to our work, with 26 coffee batches taken from several locations worldwide, using SPME-GC-MS as the analytical technique for volatile compounds, but analysed samples as bulk roasted coffee, while we analysed almost 250 individual bean samples. de Toledo et al. (2016) used literature data of volatile compounds from coffees roasted at different roasting degrees, to build a discriminant analysis (DA) model to classify for the thermal treatment applied. The authors standardised the concentrations of volatile compounds between different studies by taking pyridine as a “reference”, and selected five volatiles, mostly pyrazines, for statistics. However from our data, it is shown that pyridine is not the compound with the lowest variation among samples. In our case, the eigenvalues obtained from the PCA demonstrated that pyridine, 2-methyl-pyrazine, acetic acid, furfural and 5-methylfurfural were the volatile compounds explaining most of the observed variance in our dataset.

Our results demonstrate that coffee volatile compounds can be used as indicators for geographical origin. This information could offer indication that, despite the single bean variability found in terms of volatile compounds, there is still enough compositional difference among batches coming from different origins. This is not just due to the species differences, but probably linked to compositional differences arising from different agronomical and post-harvest processing. In addition, these results might be useful for authenticity purposes, for example to identify adulteration, and can be used in addition to rapid methods that target specific volatile compounds.

Despite the observed intra-batch variability – which is likely to be due to variations in sunlight, soil characteristics, plant-to-plant differences, and different ripening degree even on the same plant – it is still possible to detect significant differences in the volatile profiles of coffee beans coming from different regions. This study aims to obtain a broad picture of products available on the market, so that the industry could benefit from the understanding of the degree of variation in coffee volatile composition, and also reporting that good geographical discrimination can be achieved by exclusively using volatile compound analysis.

Considering the high variability found on the market, is it suggested that future research should be focused on achieving higher product consistency, starting from optimisation of the agronomic practices to obtain green beans with lower compositional variability, or to optimise the processing conditions, when methods able to rapidly discriminate the coffee beans in a non-destructive manner would be available. In the latter case, variability of coffee aroma would represent an opportunity for the coffee industry to expand the range of flavours by obtaining different aroma profiles starting from the same production batch.

## 4 Conclusions

This paper reported on the analysis of single coffee beans using SPME-GC-MS to study the variation of volatile compounds within and between batches of coffee beans roasted under the same conditions and analysed individually. This is the first report on the variability of aroma compounds formed in single coffee beans analysed by SPME-GC-MS.

The inter-batch variability was higher than the intra-batch variability for all volatile compounds studied, while our results demonstrate that the single bean variability can reach up to 179% CV for some compounds. The most variable compounds were 2,3-butanediol, 3-ethylpyridine and hexanal. As these compounds are potent odorants, this information might have a practical interest in showing the variation expected from single beans, with consequences in terms of standardisation of final coffee aroma. On the contrary, other compounds such as 2-furanmethanol, 1-(acetyloxy)-2-propanone and 2-formyl-1-methylpyrrole are the most consistent ones, both within- and among-batches (CV ~ 15–20%). In addition, we found that phenols and heterocyclic nitrogen compounds are the chemical groups showing the highest intra-batch variation, especially in some samples where values above 100% (CV) were found, while ketones were the most uniform compounds, with CV below 20% for all 25 batches.

Data on volatile compound variation of commercial samples could be used to develop a fundamental understanding of the relationship between green coffee composition and volatile compounds of the resulting roasted coffee, as well as to obtain products with more consistent quality. Despite the high variability found, we have shown that single bean volatile composition can be effectively used as a valid indicator of the coffee origin to build reliable classification models.

Further research could apply different roasting conditions or could build prediction models based on specific coffee volatile compounds, especially targeting select compounds, for example potent odorants.

The following is the supplementary data related to this article.

Supplementary Table 1 Volatile compounds in roasted coffee beans to show the intra-batch variation of chemical groups. Values are the average of 10 coffee beans expressed as % of the total GC peak area, followed by the standard deviation. Supplementary Table 1
