# Supplementary material for: Variability of single bean coffee volatile compounds of Arabica and robusta roasted coffees analysed by SPME-GC-MS
Source: Food Res Int. 2018 Jun;108:628–40. doi: 10.1016/j.foodres.2018.03.077 (PMC5960070; doi:10.1016/j.foodres.2018.03.077)
Supplement: Supplementary Table 1 — Volatile compounds in roasted coffee beans to show the intra-batch variation of chemical groups. Values are the average of 10 coffee beans expressed as % of the total GC peak area, followed by the standard deviation. [file mmc1.docx]

Supplementary Table 1.

Volatile compounds in roasted coffee beans to show the intra-batch variation of chemical groups. Values are the average of 10 coffee beans expressed as % of the total GC peak area, followed by the standard deviation.

| **Species** | **Proc.** | **Origin** | **Code** | **Aldehydes** | | |  | **Pyrazines** | | | **Ketones** | | | **Phenols** | | | **Acids** | | | **Heterocyclic N** | | |
| --- | --- | --- | --- | --- | --- | --- | --- | --- | --- | --- | --- | --- | --- | --- | --- | --- | --- | --- | --- | --- | --- | --- |
| Arabica | Dry | Indonesia | A | 11.34 | ± | 4.29 |  | 24.66 | ± | 7.60 | 4.08 | ± | 0.42 | 1.56 | ± | 1.17 | 16.48 | ± | 3.57 | 18.21 | ± | 5.83 |
| Arabica | Dry | Brazil | E12 | 18.21 | ± | 3.80 |  | 31.18 | ± | 4.13 | 4.39 | ± | 0.74 | 0.37 | ± | 0.10 | 17.94 | ± | 6.45 | 7.61 | ± | 1.29 |
| Arabica | Dry | Brazil | E | 12.97 | ± | 3.61 |  | 30.21 | ± | 6.07 | 4.03 | ± | 0.66 | 0.83 | ± | 0.84 | 17.27 | ± | 4.70 | 12.16 | ± | 9.09 |
| Arabica | Dry | Ethiopia | C | 18.20 | ± | 4.52 |  | 25.76 | ± | 4.56 | 4.34 | ± | 0.70 | 0.81 | ± | 0.30 | 19.16 | ± | 3.83 | 11.05 | ± | 4.98 |
| Arabica | Dry | Ethiopia | E7 | 17.87 | ± | 4.48 |  | 28.53 | ± | 4.42 | 5.17 | ± | 1.03 | 0.63 | ± | 0.15 | 17.18 | ± | 5.01 | 10.26 | ± | 2.10 |
| Arabica | Dry | Uganda | E8 | 17.62 | ± | 5.39 |  | 27.37 | ± | 9.10 | 4.77 | ± | 1.11 | 0.64 | ± | 0.47 | 19.56 | ± | 5.24 | 9.24 | ± | 2.96 |
| Arabica | Dry | Uganda | E9 | 22.69 | ± | 5.46 |  | 21.48 | ± | 6.85 | 5.16 | ± | 0.65 | 0.42 | ± | 0.09 | 21.49 | ± | 5.03 | 8.42 | ± | 2.22 |
| Arabica | Wet | Mexico | MA | 17.67 | ± | 3.46 |  | 32.92 | ± | 5.44 | 4.74 | ± | 0.38 | 0.48 | ± | 0.08 | 19.59 | ± | 3.13 | 6.80 | ± | 1.49 |
| Arabica | Wet | Costa Rica | Su | 17.18 | ± | 3.03 |  | 30.95 | ± | 5.09 | 4.78 | ± | 0.32 | 0.37 | ± | 0.08 | 19.97 | ± | 2.21 | 7.65 | ± | 0.83 |
| Arabica | Wet | Colombia | D | 18.69 | ± | 6.65 |  | 28.66 | ± | 6.86 | 4.46 | ± | 0.56 | 0.77 | ± | 0.40 | 17.90 | ± | 3.31 | 9.43 | ± | 4.96 |
| Arabica | Wet | Guatemala | E11 | 20.84 | ± | 2.86 |  | 26.78 | ± | 3.70 | 4.33 | ± | 0.57 | 0.42 | ± | 0.08 | 20.51 | ± | 4.95 | 7.79 | ± | 1.01 |
| Arabica | Wet | Guatemala | E5 | 16.83 | ± | 3.11 |  | 30.67 | ± | 4.35 | 4.67 | ± | 0.50 | 0.70 | ± | 0.19 | 18.12 | ± | 3.42 | 9.37 | ± | 1.10 |
| Arabica | Wet | Nicaragua | E3 | 14.23 | ± | 2.22 |  | 31.87 | ± | 5.75 | 4.10 | ± | 0.46 | 0.69 | ± | 0.14 | 18.72 | ± | 3.51 | 9.41 | ± | 1.49 |
| Arabica | Wet | Honduras | G | 18.62 | ± | 3.37 |  | 25.38 | ± | 4.53 | 4.03 | ± | 0.30 | 0.53 | ± | 0.14 | 21.80 | ± | 2.34 | 7.37 | ± | 1.95 |
| Arabica | Wet | Kenya | F | 15.71 | ± | 2.44 |  | 27.25 | ± | 5.07 | 3.78 | ± | 0.25 | 0.48 | ± | 0.09 | 22.20 | ± | 3.58 | 8.96 | ± | 1.44 |
| Arabica | Wet | Kenya | GlAr | 21.87 | ± | 3.17 |  | 22.42 | ± | 2.37 | 5.19 | ± | 0.22 | 0.71 | ± | 0.29 | 21.97 | ± | 2.38 | 8.48 | ± | 2.52 |
| Arabica | Wet | Rwanda | B | 8.62 | ± | 2.38 |  | 18.18 | ± | 4.18 | 4.39 | ± | 0.46 | 2.68 | ± | 1.05 | 14.29 | ± | 4.20 | 27.10 | ± | 8.49 |
| Arabica | Wet | India (monsoon) | E21 | 13.15 | ± | 6.45 |  | 31.42 | ± | 14.10 | 3.63 | ± | 0.90 | 1.04 | ± | 1.02 | 16.02 | ± | 6.69 | 14.87 | ± | 11.24 |
| Robusta | Wet | Guatemala | E17 | 10.15 | ± | 2.76 |  | 38.90 | ± | 7.06 | 3.91 | ± | 0.65 | 1.34 | ± | 0.28 | 13.72 | ± | 2.66 | 12.13 | ± | 1.90 |
| Robusta | Wet | Vietnam | E18 | 12.01 | ± | 4.86 |  | 40.55 | ± | 9.47 | 3.89 | ± | 0.70 | 1.22 | ± | 0.29 | 14.99 | ± | 3.38 | 9.20 | ± | 2.34 |
| Robusta | Wet | Vietnam | GlRo | 14.02 | ± | 5.96 |  | 32.95 | ± | 7.81 | 4.08 | ± | 0.63 | 1.02 | ± | 0.72 | 20.17 | ± | 3.99 | 9.21 | ± | 3.95 |
| Robusta | Wet | India | E15 | 12.90 | ± | 5.01 |  | 38.15 | ± | 9.64 | 3.31 | ± | 0.65 | 0.83 | ± | 0.21 | 16.83 | ± | 4.53 | 7.86 | ± | 2.05 |
| Robusta | Wet | India | E16 | 8.56 | ± | 2.49 |  | 41.45 | ± | 2.87 | 2.84 | ± | 0.30 | 1.13 | ± | 0.35 | 14.36 | ± | 4.32 | 11.95 | ± | 5.23 |
| Robusta | Dry | India | E20 | 12.32 | ± | 2.43 |  | 40.18 | ± | 5.59 | 3.40 | ± | 0.38 | 1.16 | ± | 0.41 | 18.01 | ± | 2.87 | 7.29 | ± | 1.51 |
| Robusta | Dry | Uganda | E19 | 11.89 | ± | 4.38 |  | 38.09 | ± | 7.89 | 3.75 | ± | 0.52 | 1.04 | ± | 0.25 | 16.46 | ± | 4.10 | 9.50 | ± | 2.94 |
